# Little Helpers

**Published:** 1871-10

**Authors:** 


### LITTLE HELPERS.

BY UNCLE BOB.

There are ever so many children in the world, yet, there are but two kinds. Shall I tell you what they are? I shall call them "little *helpers*," and "little *troublers*," and I guess that before they are ten or twelve years old, there are more "troublers" than "helpers!" What do you suppose is the reason of this?

It is because little folks are selfish, and do not think of other folks as much as they do of themselves.

I know some little boys, and girls, too, who ask their fathers and mothers to do a great many things, which they ought to do for themselves. They are getting to be big, too, but they let others wait on them. Now, I believe that any little folks who can read this, can learn to do something, right away, which they have never been in the habit of doing. Let me ask you some questions: Do you put on your own stockings and shoes; dress yourself; wash your face and hands; and brush your hair and teeth every day? Do you try to be careful at the table and not make trouble when you eat? If you do, you do a great deal that is a help to your mother, for these things you must know, are what you can learn to do very soon, if you try.

Do you remember to shut the doors after you when you go through the house? Do you try to see if there are any errands you can do for some of the grown-up people, or do you forget to do errands when they are given to you? Now, these are all little things, but if you do them nicely, you are "little helpers." You help your mother, or some one else, a great deal, when you try to be thoughtful.

Now, you may be a little troubler in a great many ways, and these are some of them: If you forget to do what you have been told, or are cross when doing it. If you are careless and do not try to be neat and clean. Or, if you do not try to take care of yourself, and must have some one wash, dress, or even tell you to be ready in time.

Some time when you come home and find that mother is tired and worried because things don't get along easy, you just stop and think what you can do! See if you can't do some little thing for her. Run out and get some kindlings, perhaps, for the fire, or take your little sister or brother, so that he can't tease mama. You don't know how happily she will say to you, "yes, my little dear, you *are* mama's little 'helper' to-day."

You will feel a great deal happier, than if you had not tried to do something for *her*, rather than for yourself.

A great many boys and girls don't know how much they *can* do. It is easy for very little folks to *do* a great deal. And when there is nothing for you to do, your play will be a great deal more fun for you, than it would have been if you had not been doing something for some one else.

Here is another thing for you to try and not do. Something which will make you manly, or lady-like, if you are a little girl. See if you can not get through a whole day without crying. If you happen to fall or get a bump on your head, don't begi to cry, and don't run to mama with your face wrinkled and twisted, but just try and laugh instead of cry. You would think it funny if your big papa should lay down his tools, when at work, and run around crying and scolding. I know you would laugh at him, perhaps call him a great baby, not to rub his head or hold his finger till it stopped aching, and then go to work again.

Suppose your mama should want to use something out of her work-basket or table, and could not find it. Wouldn't it be real silly for her to sit down and commence crying?

Now, don't you see what I want you all to do? I guess you can tell. Try this very day to see what "little helpers" you can be, and don't ever be "little troublers" any more.

